# Heart-Rate-Dependent Right-to-Left Shunting Through a Patent Foramen Ovale in Severe Right Ventricular Dysfunction: A Case Report

**DOI:** 10.3390/jcm15166188

**Published:** 2026-08-10

**Authors:** Qianfeng Xiao, Xin Wei, Ying Xu, Si Wang

**Affiliations:** Department of Cardiology, West China Hospital, Sichuan University, 37 Guoxue Road, Chengdu 610041, China; xiaoqf@wchscu.cn (Q.X.); weixin@wchscu.cn (X.W.); xy_123456@wchscu.cn (Y.X.)

**Keywords:** inflammatory cardiomyopathy, refractory hypoxemia, patent foramen ovale, right-to-left shunt, biventricular output mismatch, right ventricular dysfunction

## Abstract

**Background:** Right-to-left shunting through a patent foramen ovale (PFO) is an underrecognized yet potentially reversible cause of refractory hypoxemia, particularly in patients with right ventricular dysfunction. This case report describes heart-rate-dependent right-to-left shunting through a PFO causing refractory hypoxemia in a patient with inflammatory cardiomyopathy and severe right ventricular dysfunction, presumably arising from biventricular output mismatch. **Case Presentation:** We report the case of a 41-year-old male with inflammatory cardiomyopathy and a recently implanted single-chamber pacemaker (VVI mode, lower rate limit 50 bpm), admitted for decompensated heart failure. After initial clinical improvement with guideline-directed therapy, the patient’s intrinsic heart rate declined, and ventricular pacing at 50 bpm became the dominant rhythm. He subsequently developed refractory hypoxemia unresponsive to mechanical ventilation. Systematic hemodynamic assessment was performed using transthoracic echocardiography and thoracic electrical bioimpedance (TEB) monitoring at different pacing rates. **Results:** Echocardiographic evaluation revealed dynamic interatrial shunting through a PFO with the following characteristics: left-to-right at a pacing rate of 80 bpm and right-to-left at 50 bpm. Hemodynamic and echocardiographic data suggested that bradycardia induced biventricular output mismatch—left ventricular outflow tract velocity–time integral (VTI) increased by approximately 38% (from 17.5 cm to 24.1 cm), whereas right ventricular outflow tract VTI increased by only approximately 4% (13.3 cm vs. 13.8 cm). This mismatch likely resulted in relative elevation of right atrial pressure, thereby driving right-to-left shunting through the PFO. Increasing the pacing rate to 80 bpm reversed the shunt direction, normalized oxygenation, and facilitated successful extubation. **Conclusions:** This case suggests that in patients with severe right ventricular dysfunction, bradycardia may induce biventricular output mismatch with substantially greater left than right ventricular stroke volume augmentation, and presumably relative elevation of right atrial pressure, potentially leading to dynamic right-to-left shunting through a PFO. For such patients with unexplained hypoxemia, the possibility of dynamic PFO shunting should be considered. Appropriately increasing the pacing rate may help restore biventricular output matching, reverse shunt direction, and improve oxygenation; individualized heart rate management strategies warrant clinical consideration.

## 1. Introduction

Right-to-left shunting through a patent foramen ovale (PFO) is an underrecognized yet potentially reversible cause of refractory hypoxemia. PFO is present in approximately 25% of the general population [1], and most remain clinically silent; however, under specific conditions, clinically significant right-to-left shunting may occur. Recognized mechanisms leading to PFO-associated hypoxemia can be broadly classified into two categories: the first involves reversal of the transatrial pressure gradient, including sustained elevation of right atrial pressure (e.g., pulmonary hypertension, right heart failure) and transient pressure fluctuations (e.g., functional triggers such as obstructive sleep apnea or pregnancy) [2,3,4]; the second involves non-pressure-dependent flow redirection, whereby venous blood is preferentially directed toward the foramen ovale due to anatomical variants or tricuspid regurgitation jets impinging directly on the interatrial septum [5,6,7]. However, heart-rate-dependent dynamic PFO shunt reversal in the setting of severe right ventricular dysfunction has rarely been reported.

We report the case of a young male patient with inflammatory cardiomyopathy and an implanted single-chamber pacemaker, who developed life-threatening hypoxemia due to heart-rate-dependent right-to-left shunting through a previously occult PFO in the setting of severe right ventricular dysfunction. Through real-time echocardiography and non-invasive hemodynamic monitoring at different pacing rates, we elucidated the mechanism of this heart rate-dependent shunt reversal and discussed the clinical implications of individualized heart rate management in patients with right heart failure.

## 2. Case Report

### 2.1. Clinical Presentation

A 41-year-old male was admitted to our hospital for decompensated heart failure, presenting with decreased urine output for 1 week and dyspnea for 3 days. The patient had been diagnosed with HIV infection 2 years prior and had been receiving antiretroviral therapy since diagnosis with dolutegravir (50 mg/day, oral) and albuvirtide (320 mg/week, intravenous).

The patient had a complex recent medical history with multiple hospitalizations. Ten months before the current admission, he was diagnosed with inflammatory myopathy manifesting as muscle weakness and ptosis, and was treated with intravenous immunoglobulin (IVIG) and glucocorticoids, with oral prednisone (20 mg/day) maintained after discharge. Seven months prior, the patient was hospitalized for acute myocarditis; cardiac magnetic resonance (CMR) imaging demonstrated patchy subepicardial prolonged T2 signal in the basal and mid interventricular septum and subepicardial late gadolinium enhancement (LGE) in the interventricular septum and left ventricular free wall, fulfilling the updated Lake Louise criteria for myocardial inflammation [8]. Endomyocardial biopsy was recommended but declined by the patient and family. He received intravenous glucocorticoid therapy followed by maintenance oral prednisone (tapered to 5 mg/day). Approximately 1 month before the current admission, the patient developed atrial fibrillation (AF) with complete (third-degree) atrioventricular block (AVB) and elevated myocardial injury markers, prompting intensification of glucocorticoid therapy. While myocardial injury markers declined with treatment, the AVB did not resolve, and a single-chamber permanent pacemaker was implanted (VVI mode, lower rate limit 50 bpm), with prednisone adjusted to 40 mg/day. After discharge, the patient self-discontinued glucocorticoid therapy.

**Figure 1:**
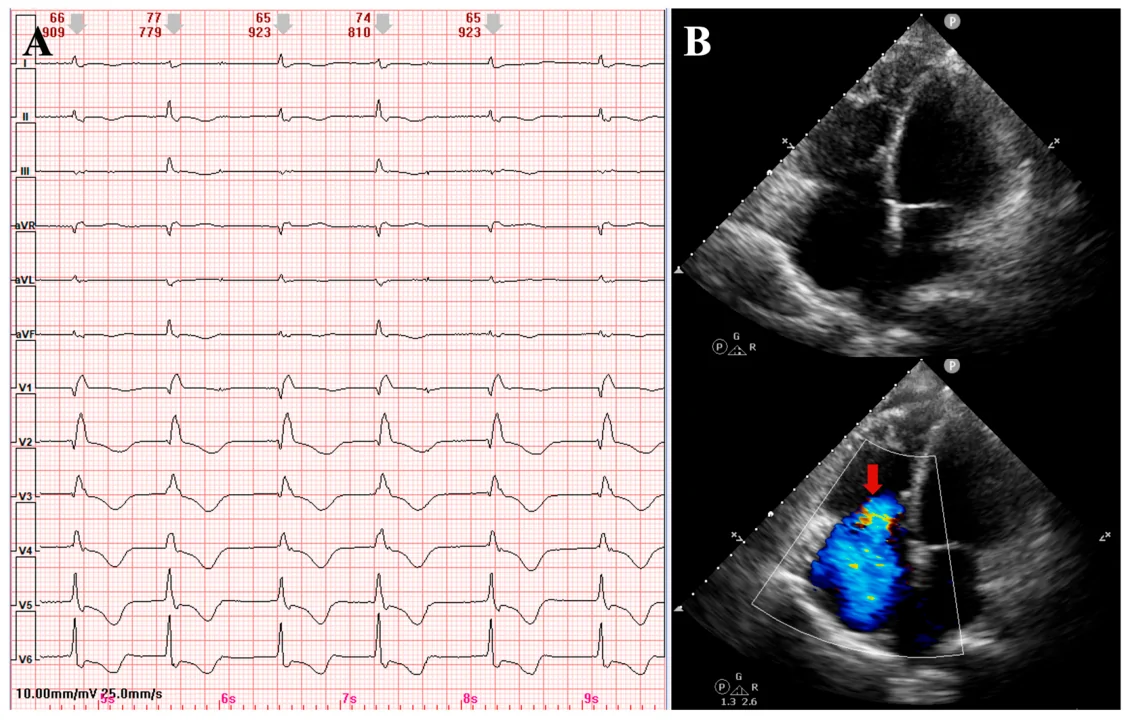
Clinical findings at admission. (**A**) Electrocardiogram showing atrial fibrillation with a ventricular rate of 67 bpm, low voltage in the limb leads, and complete right bundle branch block. (**B**) Transthoracic echocardiography (upper panel: apical four-chamber view; lower panel: color Doppler) demonstrating four-chamber dilatation, biventricular systolic dysfunction, and severe functional tricuspid regurgitation (red arrow).

On admission, physical examination revealed a blood pressure of 105/62 mmHg, an intrinsic heart rate of 75 bpm, an irregular rhythm with a grade 2/6 systolic murmur at the tricuspid area, bilateral basal crackles, mild lower extremity edema, and oxygen saturation of 100% on nasal cannula oxygen (2 L/min). Electrocardiography (ECG) demonstrated AF with a ventricular rate of 67 bpm, low voltage in the limb leads, and complete right bundle branch block (Figure 1A). Transthoracic echocardiography (TTE) showed four-chamber dilatation with biventricular systolic dysfunction: left ventricular end-diastolic diameter (LVEDD) 55 mm, left ventricular ejection fraction (LVEF) 36%, and tricuspid annular plane systolic excursion (TAPSE) 12 mm; severe functional tricuspid regurgitation was present without evidence of pulmonary hypertension (Figure 1B). Laboratory investigations revealed high-sensitivity troponin T (hs-TnT) of 1255.0 ng/L and N-terminal pro-B-type natriuretic peptide (NT-proBNP) of 15,395 ng/L. HIV viral load was well suppressed (<20 copies/mL), with a CD4+ T-cell count of 251 cells/μL. Comprehensive screening for opportunistic infections was negative.

### 2.2. Initial Treatment and Clinical Course

Given the clinical presentation of severe inflammatory cardiomyopathy with markedly elevated myocardial injury markers, likely precipitated by abrupt discontinuation of glucocorticoids, and the inadequacy of previous conventional-dose regimens to prevent recurrent flares, high-dose intravenous methylprednisolone (240 mg/day) was initiated with reference to current guidelines [9], following multidisciplinary consultation with the infectious diseases department and informed consent, with continued antiretroviral therapy coverage. Concurrent therapy included intravenous diuretics, and empiric antibiotics. After 1 week, cardiac biomarkers showed a significant declining trend (hs-TnT decreased to 118.6 ng/L, NT-proBNP decreased to 3910 ng/L), suggesting effective control of myocardial inflammation.

**Figure 2:**
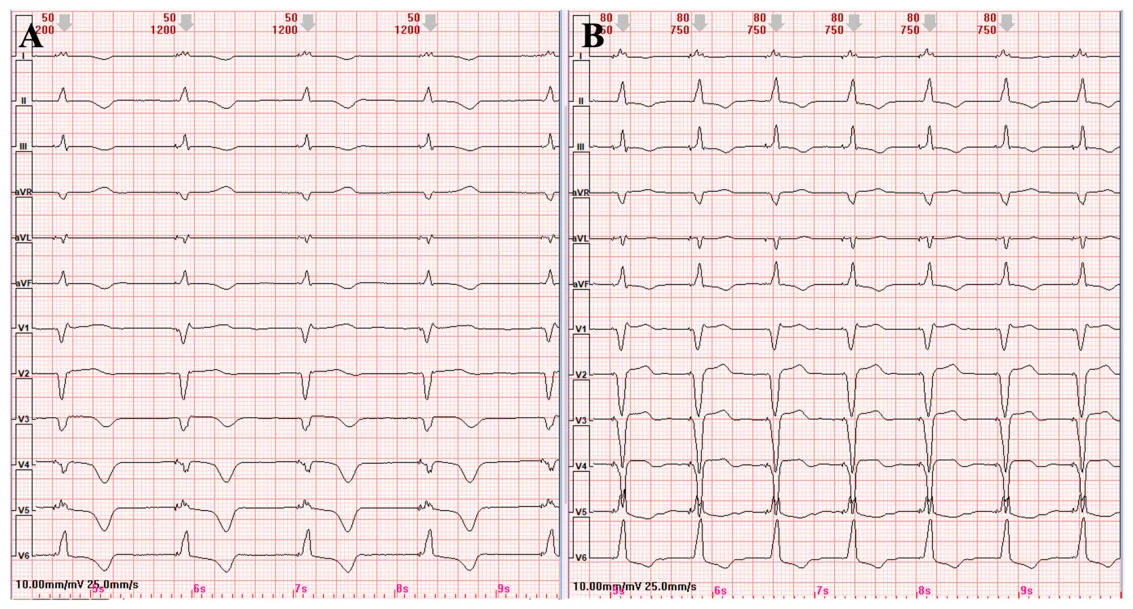
Electrocardiograms at different pacing rates. (**A**) VVI pacing at 50 bpm as the dominant rhythm. (**B**) After pacemaker reprogramming to 80 bpm.

**Figure 3:**
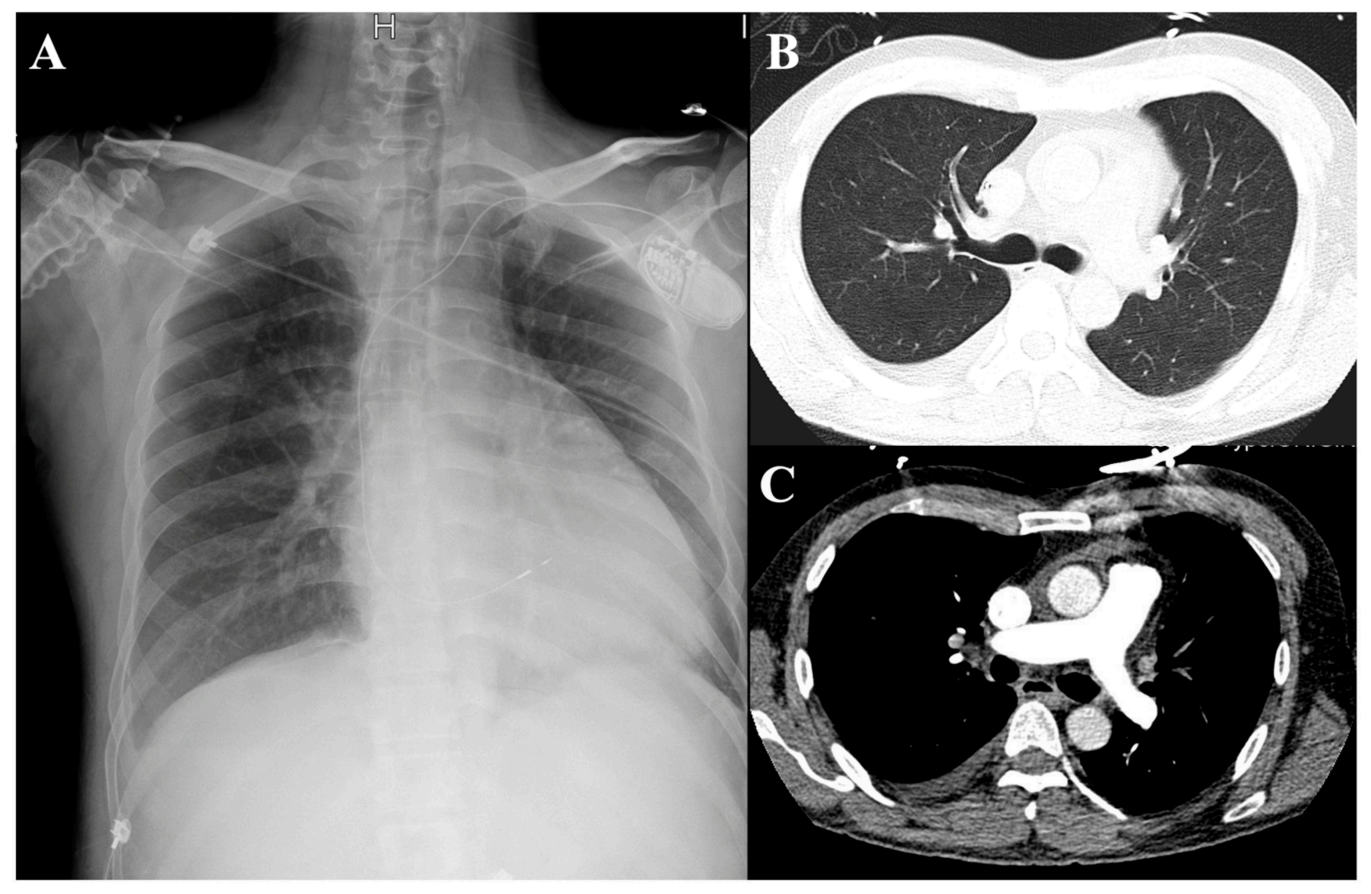
Imaging evaluation of hypoxemia etiology. (**A**) Chest radiograph showing no acute pulmonary edema. (**B**) Chest CT showing no significant pulmonary infection. (**C**) CT pulmonary angiography excluding pulmonary embolism.

During this period, as the patient’s intrinsic heart rate declined, ventricular pacing at 50 bpm became the dominant rhythm (Figure 2A). Concurrently, the patient developed progressive hypoxemia poorly responsive to non-invasive positive pressure ventilation (expiratory positive airway pressure [EPAP] 8–10 cmH_2_O), with PaO_2_/FiO_2_ progressively declining to 50 mmHg. Endotracheal intubation and invasive mechanical ventilation (positive end-expiratory pressure [PEEP] 6–8 cmH_2_O) yielded only modest improvement (PaO_2_/FiO_2_ approximately 100 mmHg). Follow-up chest radiography and computed tomography (CT) revealed no acute pulmonary edema or significant pulmonary infection (Figure 3A,B), and CT pulmonary angiography (CTPA) excluded pulmonary embolism (Figure 3C).

### 2.3. Hemodynamic Assessment and Diagnosis

Given the clear temporal association between heart rate decline and worsening hypoxemia, the pacing rate was increased to 80 bpm (Figure 2B). Oxygenation improved rapidly (PaO_2_/FiO_2_ rose to 225 mmHg).

**Figure 4:**
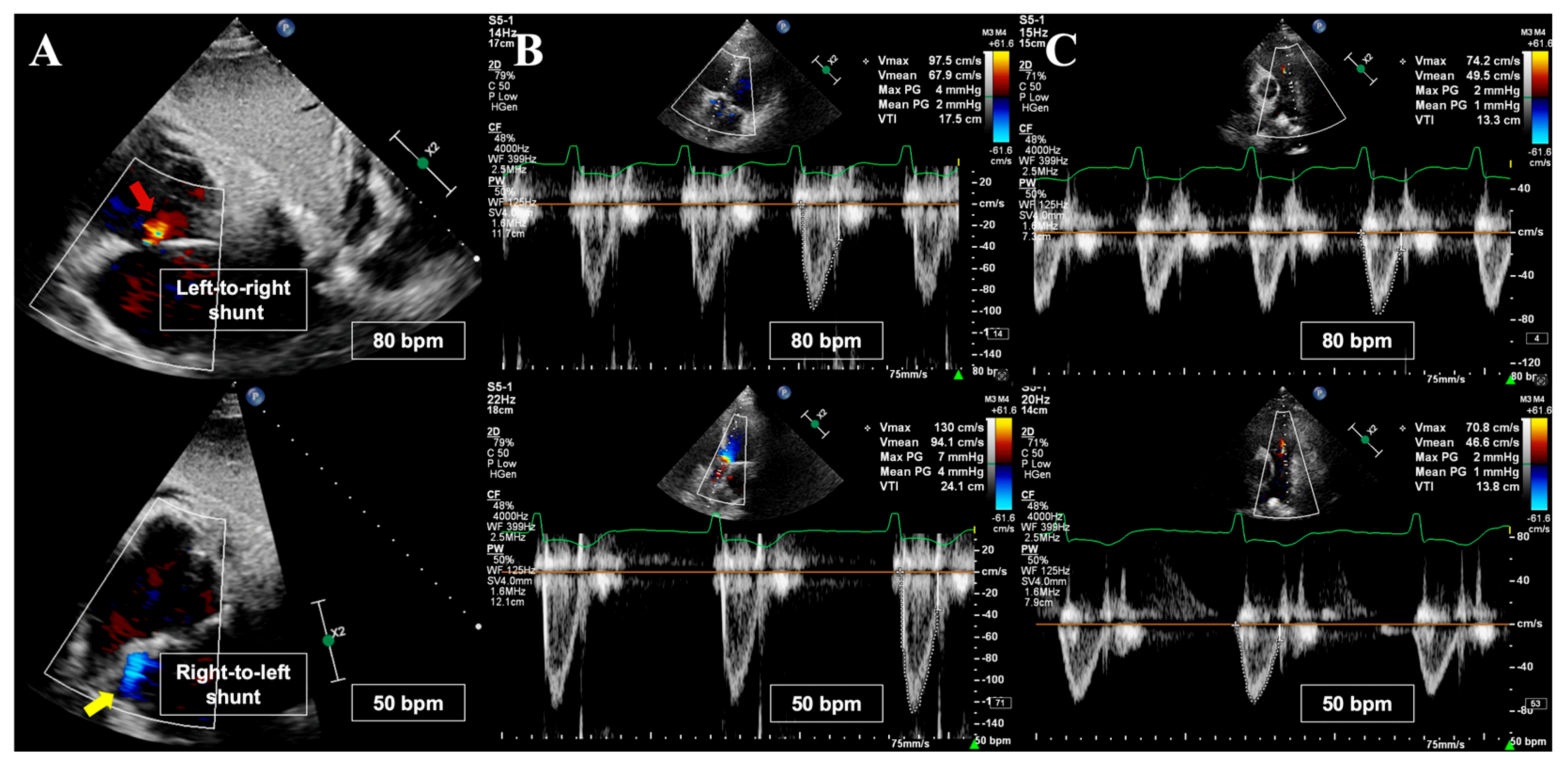
Heart rate-dependent interatrial shunting and biventricular hemodynamic mismatch. (**A**) Transthoracic echocardiography demonstrating dynamic shunting through a PFO: left-to-right at 80 bpm (upper panel, red arrow) and right-to-left at 50 bpm (lower panel, yellow arrow). (**B**) Pulsed-wave Doppler of the left ventricular outflow tract (LVOT, diameter 20 mm) showing VTI of 17.5 cm at 80 bpm (upper) and 24.1 cm at 50 bpm (lower); calculated stroke volume 55.0 mL and 75.7 mL, cardiac output 4.4 L/min and 3.8 L/min, respectively. (**C**) Pulsed-wave Doppler of the right ventricular outflow tract (RVOT, diameter 23 mm) showing VTI of 13.3 cm at 80 bpm (upper) and 13.8 cm at 50 bpm (lower); calculated stroke volume 55.2 mL and 57.3 mL, cardiac output 4.4 L/min and 2.9 L/min, respectively.

Repeat TTE demonstrated dynamic interatrial shunting through a PFO: at a pacing rate of 80 bpm, color Doppler showed left-to-right shunting (Figure 4A, upper panel; Supplementary Video S1); when the rate was reduced to 50 bpm, the shunt reversed to right-to-left (Figure 4A, lower panel; Supplementary Video S2).

**Figure 5:**
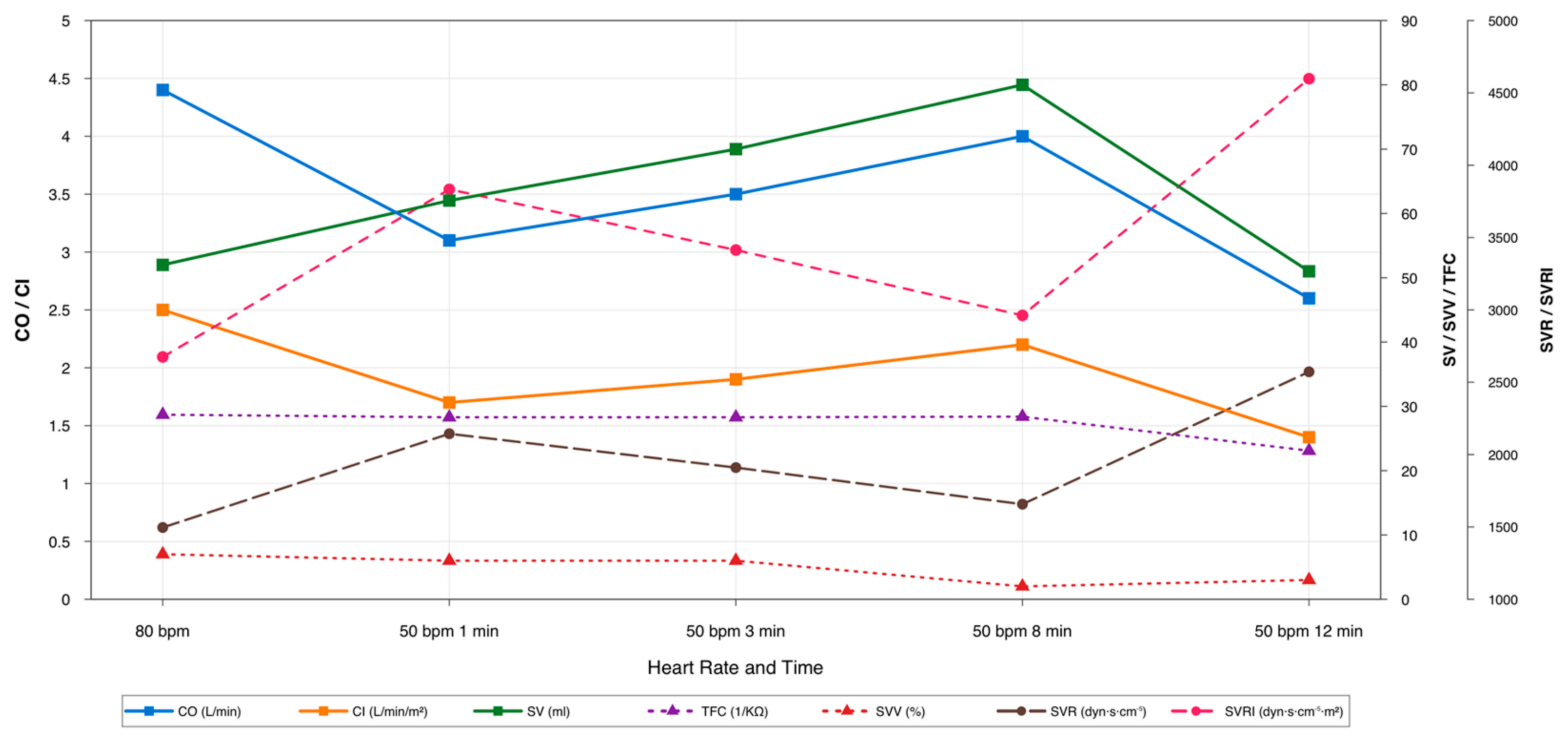
Non-invasive hemodynamic monitoring at different pacing rates. Hemodynamic data obtained using thoracic electrical bioimpedance (TEB). Following reduction in the pacing rate from 80 to 50 bpm, SV initially increased but declined to baseline by 12 min, with CI falling to 1.4 L/min/m^2^. CO = cardiac output; CI = cardiac index; SV = stroke volume; TFC = thoracic fluid content; SVV = stroke volume variation; SVR = systemic vascular resistance; SVRI = systemic vascular resistance index.

Continuous non-invasive hemodynamic monitoring using thoracic electrical bioimpedance (TEB; BioZ-2011, Med-Med Medical Equipment Co., Ltd., Shenzhen, China) was performed to assess hemodynamic trends during this process (Figure 5) [10,11]. At a pacing rate of 80 bpm, stroke volume (SV) was 52 mL with a cardiac index (CI) of 2.5 L/min/m^2^. When the pacing rate was reduced to 50 bpm, SV initially increased, partially compensating for the lower heart rate. However, this response was unsustained, with SV returning to 51 mL and CI declining to 1.4 L/min/m^2^ by 12 min.

Doppler echocardiographic assessment revealed that during bradycardia, left ventricular outflow tract (LVOT) velocity–time integral (VTI) increased compensatorily (from 17.5 cm at 80 bpm to 24.1 cm at 50 bpm) (Figure 4B), whereas right ventricular outflow tract (RVOT) VTI remained essentially unchanged (13.3 cm at 80 bpm vs. 13.8 cm at 50 bpm) (Figure 4C). Calculated cardiac output from LVOT and RVOT (diameters 20 mm and 23 mm, respectively) was matched at 80 bpm but diverged markedly at 50 bpm (3.8 vs. 2.9 L/min). TR severity was similar at both pacing rates, and the TR jet was not directed toward the interatrial septum, arguing against a flow-driven shunt mechanism. This biventricular output mismatch presumably resulted in a relative elevation of right atrial pressure, potentially driving right-to-left shunting through the PFO. Upon restoration of the pacing rate to 80 bpm, the shunt reverted to left-to-right.

### 2.4. Treatment and Outcome

Based on the above hemodynamic and imaging findings, the pacemaker lower rate limit was programmed to 80 bpm. This intervention reversed the right-to-left shunt, normalized systemic oxygenation, and facilitated successful liberation from mechanical ventilation. Following extubation, contrast echocardiography (agitated saline study) was performed at a pacing rate of 80 bpm, with agitated saline injected via the left antecubital vein. At rest, microbubbles appeared in the left heart by the 3rd cardiac cycle after right heart opacification, with significant opacification by the 6th cardiac cycle, persisting for more than 10 cardiac cycles. Following a Valsalva maneuver, massive left heart opacification occurred immediately upon release (Supplementary Videos S3 and S4), further confirming the anatomical substrate for PFO-mediated right-to-left shunting.

The patient was discharged after stabilization. During follow-up, glucocorticoid therapy was tapered and discontinued, and the patient is currently receiving guideline-directed medical therapy with continued antiretroviral therapy and a pacing rate of 80 bpm. The clinical course has remained stable without rehospitalization, with no re-elevation of hs-TnT and no interatrial shunting on repeat TTE.

## 3. Discussion

This case illustrates a rare but clinically significant proposed mechanism of refractory hypoxemia: heart-rate-dependent dynamic right-to-left shunting through a PFO in the setting of severe right ventricular dysfunction, presumably driven by biventricular output mismatch. Several pathophysiological and clinical insights can be derived from this case.

First, the mechanism of hypoxemia in this patient differs from conventionally described PFO-associated hypoxemia. Right-to-left shunting through a PFO is typically driven by sustained elevation of right atrial pressure in the setting of pulmonary hypertension or chronic right heart failure [1,2], or by anatomical flow redirection as seen in platypnea–orthodeoxia syndrome [5,6]. A recent case report also described a “flow-driven” mechanism in which a massive tricuspid regurgitation jet directed blood across the interatrial septum via the Coandă effect, maintaining right-to-left shunting even in the absence of elevated right atrial pressure [7]. However, the mechanism in the present case was distinctly rate-dependent: Doppler data suggested that the failing right ventricle was unable to match the compensatory increase in left ventricular output during bradycardia, thereby presumably creating the hemodynamic conditions for shunt reversal. This suggests that in patients with implanted cardiac pacemakers and unexplained hypoxemia, rate-dependent hemodynamic changes should be considered, and comprehensive echocardiographic evaluation may be valuable in identifying the underlying mechanism [12].

Second, the biventricular output mismatch inferred from this case provides further pathophysiological considerations. In patients with severe right ventricular dysfunction, cardiac output is highly heart rate-dependent because the failing right ventricle has limited capacity to augment SV during bradycardia. This observation is consistent with the hemodynamic findings described by Goldstein et al. in right ventricular infarction—the failing right ventricle and the preload-deprived left ventricle exhibit relatively fixed SV, rendering cardiac output highly dependent on heart rate [13]. When heart rate decreased from 80 bpm to 50 bpm, LVOT VTI increased by approximately 38%, reflecting preserved left ventricular Frank–Starling reserve. In contrast, RVOT VTI increased by only approximately 4%, indicating that the dysfunctional right ventricle was unable to proportionally augment its SV. This relative imbalance between left and right ventricular output presumably resulted in relative elevation of right atrial pressure, potentially contributing to right-to-left shunting. Previous studies have confirmed that in patients with right heart failure and tricuspid regurgitation, increasing the pacing rate from below 70 bpm to 90 bpm can significantly increase cardiac output and reduce central venous pressure [14]. Furthermore, recent invasive hemodynamic evidence has demonstrated that severe bradycardia can exacerbate pulmonary circulatory pressures, and targeting heart rate escalation to 90 bpm may help reduce mean pulmonary artery pressure [15]. These findings collectively support heart rate optimization as a therapeutic target in patients with right ventricular dysfunction.

Third, positive-pressure mechanical ventilation may have influenced the hemodynamic status. Positive-pressure ventilation increases right ventricular afterload through elevation of pulmonary vascular resistance while simultaneously reducing right ventricular preload by impeding venous return [16,17]. In patients with acute right heart failure, inappropriate positive-pressure ventilation may further impair right ventricular function [18]. In the present case, invasive mechanical ventilation yielded only modest oxygenation improvement (PaO_2_/FiO_2_ approximately 100 mmHg). The lower level of PEEP applied during invasive ventilation (6–8 cmH_2_O) compared with EPAP during prior non-invasive ventilation (8–10 cmH_2_O) may have partially attenuated the compressive effect of positive pressure on the right heart system, but was insufficient to address the fundamental problem of rate-dependent biventricular mismatch.

Fourth, interventional strategies for this type of hypoxemia require consideration within the specific hemodynamic context. Percutaneous PFO closure is effective for most cases of refractory hypoxemia caused by persistent right-to-left shunting [1,19]. However, during acute decompensation of right heart failure, the PFO may serve as a physiological “pressure relief valve.” Closing the PFO during this phase could eliminate this decompressive mechanism and precipitate acute exacerbation of right ventricular afterload and right heart failure. Previous literature has reported acute hemodynamic deterioration and procedural abort during attempted PFO closure in patients with right heart failure [20]. Therefore, in the present case, increasing the pacing rate addressed the core mechanism of biventricular mismatch, restoring right ventricular forward output and correcting the shunt, while avoiding the risks of PFO closure during the acute phase [21].

Fifth, this case also has implications for heart rate management in patients with heart failure with reduced ejection fraction (HFrEF) and concomitant right ventricular dysfunction. Current guidelines recommend beta-blockers for heart rate control in HFrEF patients largely based on evidence from populations with predominantly left ventricular dysfunction. However, the impact of heart rate reduction on patients with concomitant right ventricular dysfunction remains insufficiently studied. This case suggests that right ventricular function should be carefully evaluated in HFrEF patients, as it may affect the tolerability and appropriateness of standard heart rate reduction strategies, and that a more tailored approach to heart rate targets may be warranted in this population.

Finally, several limitations should be acknowledged. First, right heart catheterization was not performed due to the patient’s critical condition. Right atrial pressure elevation as the driving force for shunt reversal was therefore inferred from non-invasive data rather than directly measured, which limits the strength of the mechanistic conclusions. Second, transesophageal echocardiography (TEE) was not performed during the critical phase; it would have provided more precise anatomical characterization of PFO size and morphology. Third, although TEB has been validated for monitoring trends in cardiac output [10,11], its absolute accuracy compared with invasive methods is limited. Fourth, contrast echocardiography was performed only at 80 bpm; it was not repeated at 50 bpm due to concern for clinical deterioration. Fifth, the optimal long-term pacing strategy and prognosis for patients with concurrent severe inflammatory cardiomyopathy and PFO-mediated shunting require further clinical follow-up.

## 4. Conclusions

This case suggests that in patients with severe right ventricular dysfunction, bradycardia may induce heart-rate-dependent right-to-left shunting through a PFO, presumably through biventricular output mismatch with substantially greater left than right ventricular stroke volume augmentation. In such patients presenting with unexplained hypoxemia, the possibility of dynamic PFO shunting should be considered. Appropriately increasing the pacing rate may restore biventricular output matching, reverse shunt direction, and improve oxygenation. This case underscores the importance of individualized heart rate management in patients with significant right ventricular dysfunction.

## Data Availability

All authors confirm that all the data that support the findings of this report are available on request from the corresponding author.

## Supplementary Materials

The following supporting information can be downloaded at: https://www.mdpi.com/article/10.3390/jcm15166188/s1, Video S1: Echocardiography of left-to-right shunting at 80 bpm; Video S2: Echocardiography of right-to-left shunting at 50 bpm; Video S3: Contrast echocardiography at rest (80 bpm); Video S4: Contrast echocardiography after Valsalva maneuver (80 bpm).
